# Radiomics parameters of epicardial adipose tissue predict mortality in acute pulmonary embolism

**DOI:** 10.1186/s12931-024-02977-x

**Published:** 2024-10-01

**Authors:** Alexey Surov, Silke Zimmermann, Mattes Hinnerichs, Hans-Jonas Meyer, Anar Aghayev, Jan Borggrefe

**Affiliations:** 1 Department of Radiology, Neuroradiology and Nuclear Medicine, Johannes Wesling University Hospital, Ruhr University Bochum, Hans-Nolte-Str. 1, 32429 Minden, Minden, Germany; 2 Institute of Laboratory Medicine, Clinical Chemistry and Molecular Diagnostics, University of Leipzig, Leipzig, Germany; 3 Clinic for Radiology and Nuclear Medicine, University Hospital Magdeburg, Magdeburg, Germany; 4 Department of Diagnostic and Interventional Radiology, University of Leipzig, Leipzig, Germany

**Keywords:** Acute pulmonary embolism, Epicardial adipose tissue, Radiomics, Mortality

## Abstract

**Background:**

Accurate prediction of short-term mortality in acute pulmonary embolism (APE) is very important. The aim of the present study was to analyze the prognostic role of radiomics values of epicardial adipose tissue (EAT) in APE.

**Methods:**

Overall, 508 patients were included into the study, 209 female (42.1%), mean age, 64.7 ± 14.8 years. 4.6%and 12.4% died (7- and 30-day mortality, respectively). For external validation, a cohort of 186 patients was further analysed. 20.2% and 27.7% died (7- and 30-day mortality, respectively). CTPA was performed at admission for every patient before any previous treatment on multi-slice CT scanners. A trained radiologist, blinded to patient outcomes, semiautomatically segmented the EAT on a dedicated workstation using ImageJ software. Extraction of radiomic features was applied using the pyradiomics library. After correction for correlation among features and feature cleansing by random forest and feature ranking, we implemented feature signatures using 247 features of each patient. In total, 26 feature combinations with different feature class combinations were identified. Patients were randomly assigned to a training and a validation cohort with a ratio of 7:3. We characterized two models (30-day and 7-day mortality). The models incorporate a combination of 13 features of seven different image feature classes.

**Findings:**

We fitted the characterized models to a validation cohort (*n* = 169) in order to test accuracy of our models. We observed an AUC of 0.776 (CI 0.671–0.881) and an AUC of 0.724 (CI 0.628–0.820) for the prediction of 30-day mortality and 7-day mortality, respectively. The overall percentage of correct prediction in this regard was 88% and 79% in the validation cohorts. Lastly, the AUC in an independent external validation cohort was 0.721 (CI 0.633–0.808) and 0.750 (CI 0.657–0.842), respectively.

**Interpretation:**

Radiomics parameters of EAT are strongly associated with mortality in patients with APE.

**Clinical trial number:**

Not applicable.

## Introduction

Acute pulmonary embolism (APE) is a potential life-threatening disorder with a high mortality [1, 2]. Therefore, an immediate diagnosis of APE is crucial. The current gold standard for the diagnosis of APE is computer tomographic pulmonary angiography (CTPA). Moreover, CTPA parameters can also predict outcomes in patients with APE. For instance, right ventricle enlargement is associated with 30-day mortality in APE [3]. Reflux of contrast medium into the inferior vena cava (IVC) is another important CTPA parameter. IVC reflux correlates with tricuspid regurgitation and with levels of troponin [4, 5]. IVC reflux also correlates with 30-day mortality [6]. Evidence suggests image analysis as an emerging diagnostic tool.

Recently, epicardial adipose tissue (EAT) has been proposed as a novel imaging biomarker in several acute conditions. In fact, EAT volume and attenuation independently distinguishes patients with and without myocardial infarction [7]. Furthermore, EAT density predicts obstructive coronary artery disease and high risk plaque features in patients with atypical chest pain [8]. Moreover, in COVID-19, EAT parameters like volume and/or density can predict adverse clinical outcomes [9]. 

In APE, previously, only one work analyzed associations between EAT and outcomes [10]. 

Currently, modern imaging post-processing methods such as radiomics are used to identify novel imaging biomarkers. For instance, in APE, several texture analysis values derived from thrombotic clots differed significantly in survivors and non-survivors [10]. 

Previously, only few studies reported the prognostic significance of radiomics-based features of EAT in several cardiac disorders [11, 12]. So far, Ilyushenkova et al. showed that the parameter „gray level non-uniformity normalized“ was an independent predictor of atrial fibrillation recurrence atrial fibrillation recurrence after catheter ablation [11]. Furthermore, radiomics signatures of EAT can also improve cardiac risk prediction in patients with coronary artery disease [12]. 

To the best of our knowledge, there are no reports about the clinical significance of radiomics-based values derived from EAT in APE. Presumably, radiomics analysis of EAT may provide novel sensitive parameters for prediction of unfavorable prognosis in APE.

The purpose of the present work was to investigate the prognostic role of radiomics-based parameters of EAT in patients with APE.

## Methods

### Data acquisition

#### Main cohort

The present retrospective study was approved by the institutional review board (Nr. 145/21, Ethics Committee, Otto-von-Guericke University of Magdeburg, Magdeburg, Germany). The study was performed in accordance with the Declaration of Helsinki. Informed consent was waived due to the retrospective design.

For this study, the clinical database was screened for cases with APE in the time-period between 2015 and 2021. Inclusion criteria for the study were:

- sufficient CTPA images at the admission of the hospital;
- available information about 30-day and 7-day mortality (according to the medical records, in all instances, the mortality was attributed to acute PE);
- thrombolytic treatment was not administered before and/or during the CTPA;
- information about troponin, lactate, sPESI and hemodynamic instability.

Exclusion criteria were as follows:

- severe image artifacts;
- missing clinical data/follow up;
- chronic PE.

**Fig. 1:**
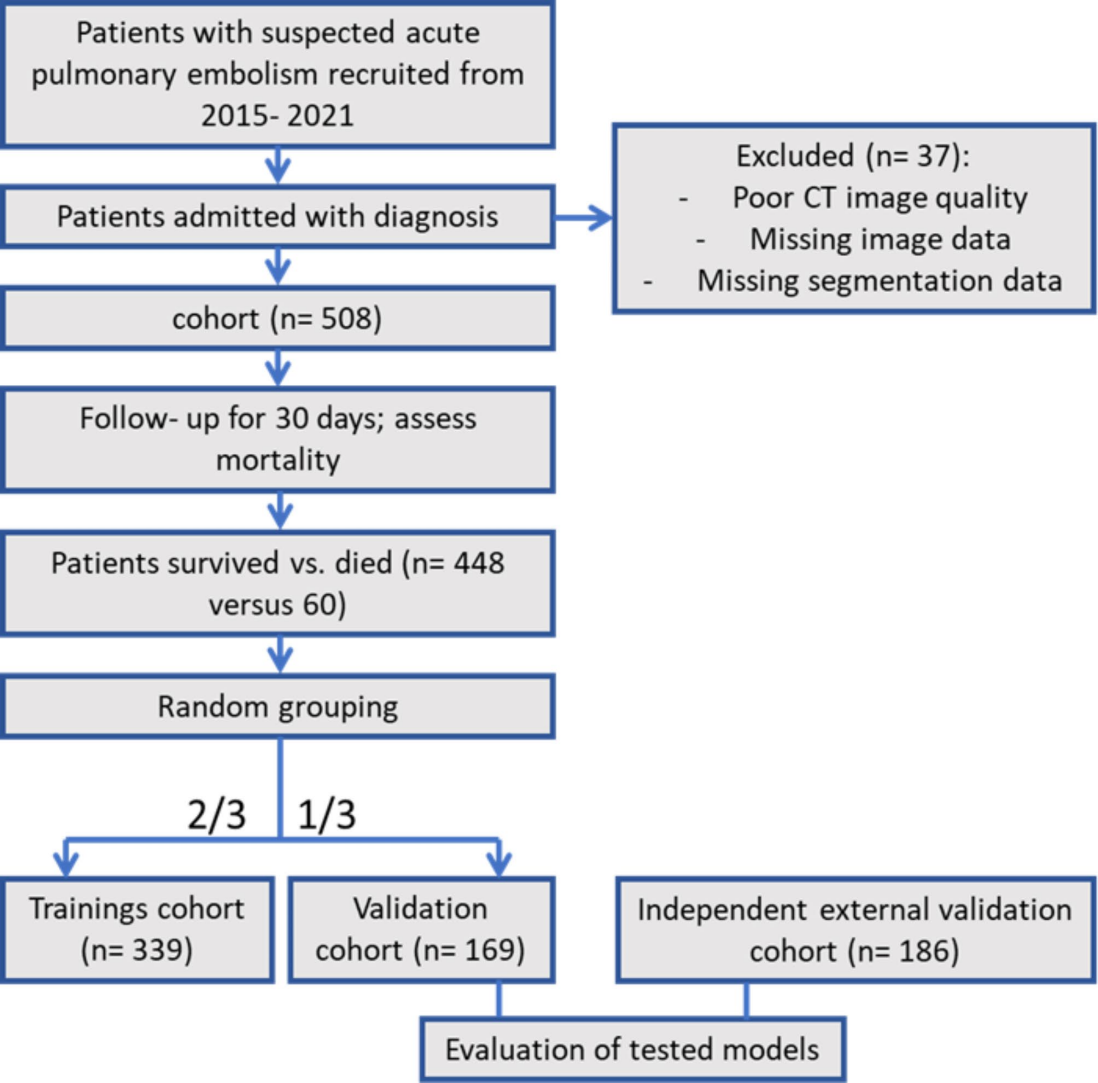
Flowchart of patient recruitment and study design

Overall, 508 patients met the inclusion criteria (Fig. 1). Patients comprised 209 females (42.1%) and 299 males (57.9%) with a mean of 64.7 ± 14.8 years.

#### Independent validation cohort

For the external evaluation, an additional cohort was collected. For this cohort, a retrospective search in the clinical database of the Department of Internal Medicine of the University of Leipzig was performed. This subproject was approved by the institutional review board (Nr. 118/19-ck, Ethics Committee, University of Leipzig, Leipzig, Germany). There were also similar inclusion and exclusion criteria. Overall, 186 patients were included (Fig. 1). Patients comprised 96 (52%) men and 90 (48%) women with a mean age of 64.1 ± 15.6 years.

### Imaging technique

CTPA was performed at admission for every patient before any previous treatment on multi-slice CT scanners (Siemens Somatom Definition AS+, Siemens Healthcare, Germany or Canon Aquilion Prime, Canon Medical Systems, Ottawara, Japan). In all cases, an iodinated contrast agent (60–150 ml Accupaque 300 mg/ml, GE Healthcare Buchler GmbH & Co. KG, Braunschweig, Germany or Imeron 300, Bracco Imaging Deutschland GmbH, Konstanz, Germany) was administrated intravenously via a peripheral venous line at a rate of 3.0–4.0 ml/s. Automatic bolus tracking was performed in the pulmonary trunk with a trigger of 100 Hounsfield units (HU). The imaging parameters were as follows: 100–120 kVp, 25–200 mAs (tube current modulated 50–400 mA), slice thickness 1 mm, and a pitch factor of 1.4.

### Analysis of EAT

**Fig. 2:**
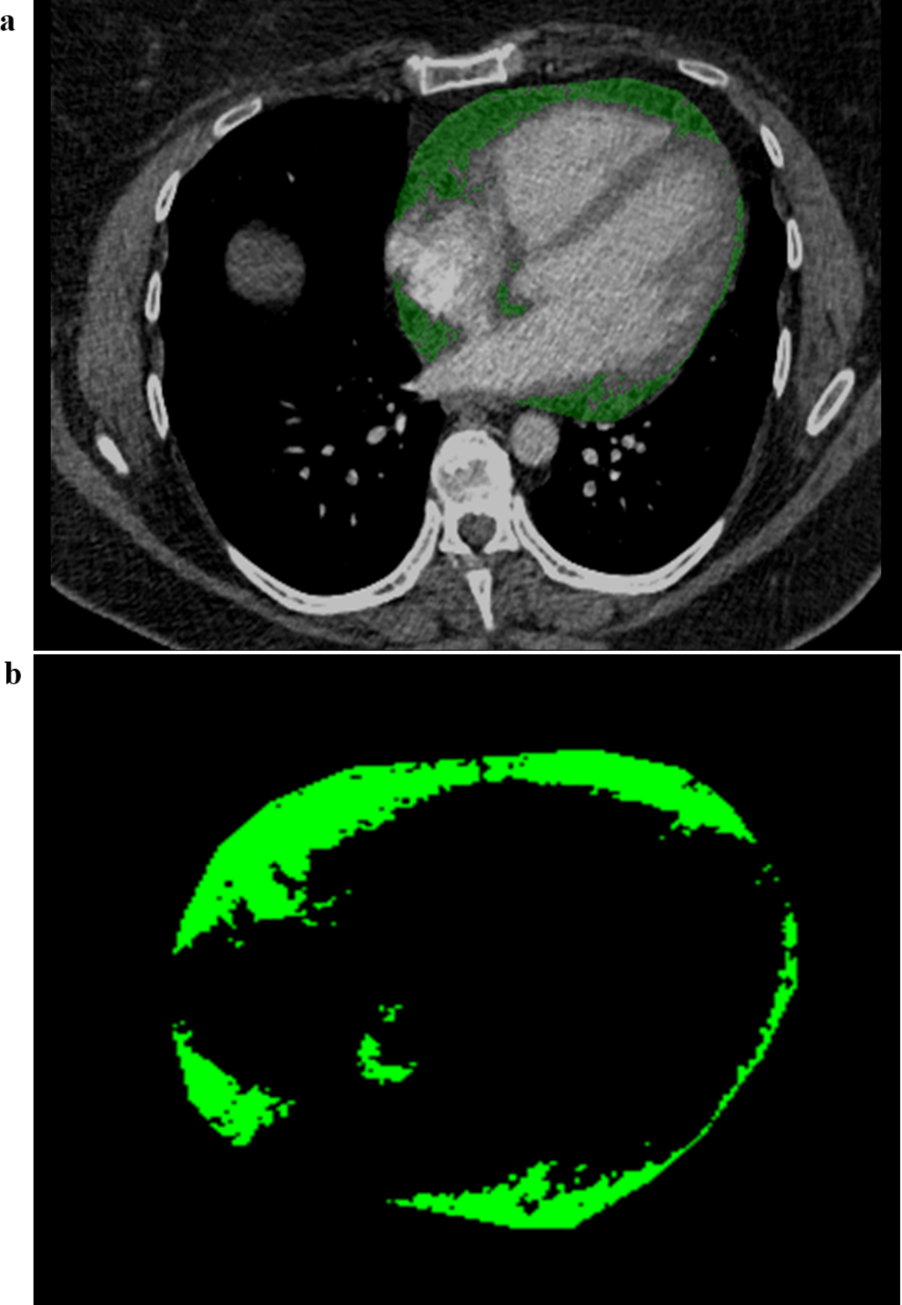
(**a**) Segmentation of EAT on CTPA. (**b**) Segmented EAT images

EAT segmentation was performed by a trained radiologist (AA). The pericardium was manually traced from the right pulmonary artery to the diaphragm respecting as anatomical limits the pulmonary artery bifurcation, the left atrium and the aortic root as the upper limit and the diaphragm and the left ventricle apex as the lower limit. Furthermore, the region of interest within the traced area was defined based on density threshold values between − 30 and − 190 Hounsfield units (HU). After three-dimensional reconstruction, EAT volume was automatically calculated by the software program. The segmentation was done on a dedicated workstation using ImageJ software (Fig. 2a). The radiologist was blinded to patient outcomes. The segmented EAT volume data were saved in DICOM format, before being transformed in NRRD format using python (Fig. 2b).

Extraction of the radiomics features was applied using the pyradiomics library (https://pyradiomics.readthedocs.io/en/latest/), a Python toolbox and used it for deep analysis of the CT data [13]. The .yaml- parameter file is stored and can be applied upon request. The datasets were normalized to -1024 HU and 1000 HU according to the pyradiomics normalization steps. We used the settings that were recommended for CT data (with customized modifications) and automatically extracted 1661 features for each patient. The results were stored in a large excel file for subsequent analysis.

Randomly, patients were assigned to a training and a validation cohort with a ratio of 7:3 (Fig. 1).

We divided the extracted features of the EAT into three main groups: (a) the first order statistics, (b) texture parameters and (c) size/shape features. The first 19 features are based on histogram and mainly describe attenuation value distribution of EAT (grey-white-level). The textural features characterize the spatial relationship between voxel grey-values and hence heterogeneity of adipose tissue measures and its coarseness, which is further described by five matrices (grey-level dependence matrix (GLDM), grey-level co-occurrence matrix (GLCM), grey-level size zone matrix (GLSZM), grey-level run-length matrix (GLRLM), and neighbourhood grey tone difference matrix (NGTDM)). Moreover, we extracted size and shape features (including EAT volume) and grey- level intensity- independent features. We applied in total seven filters for deeper image characterization: wavelet (yields eight decompositions per level in each of the three dimensions), Laplacian of Gaussian filter (emphasizes areas of grey level change), Square (takes the square of the image intensities and linearly scales them back to the original image), SquareRoot (takes the square root of the absolute image intensities and scales them back to original range), Logarithm (takes the logarithm of the absolute intesity + 1), Exponential (takes the exponential) and Gradient (returns the magnitude of the local gradient). All features were extracted from original and filtered images (shape and size features are only extracted from the original images). 

### Feature cleansing and feature selection

A correlation analysis was performed using R (version 3.5.1) for Windows. In order to account for non-normal features, we opted for Spearman correlation. We removed correlating features with correlation larger than 0.7 in order to avoid weakening of the regression model by loading variables which correlate. Further, we only used parameters which differed among the two groups (survived versus died). The SPSS modeler was used in order to further reduce feature count and ranking of features of the selection model in order to extract the key features for the model. Feature cleansing reduced the feature number to 247 features for each patient. We put the features into the random forest (RF) model, ranking the features according to their importance to the model. Based on the ranking results, we fitted the features in forward regression analysis, extracting 26 feature combination models. We selected the optimal combination (feature signature), which was able to predict the mortality of the patients. For our models, we directly implemented a logistic regression model due to its reduced small number of input features. The aim was to analyze feature combinations with minimal correlation among features.

### Statistical analysis

All statistical analyses for the present study were performed with SPSS 29.0, R (version 3.5.1) and Python (version 3.6). Mann- Whitney U test or Student’s t test was used for the continuous variables according to the test of normal distribution. The chi-square test was applied to compare categorical variables. According to the test of normal distribution, continuous variables were given as mean S ± SD or median interquartile range (IQR). Statistical significance was indicated by a two-tailed *p* value < 0.05. The receiver operating characteristic (ROC) curve was used to evaluate the performance of the models for discrimination between patients with acute pulmonary embolism that survived from non-survivors. After establishment of two suitable models, feature signatures (combinations of specific features) were validated in the validation cohort of the same cohort and in an independent external validation cohort.

## Results

Overall, 508 patients were included into the study, 209 female (42.1%), mean age, 64.7 ± 14.8 years. In the validation cohort, a total of 4.6% died within 7-days and 12.4% died within the 30-day observation study period. Table 1 provides descriptive data of the patient cohort. In all cases, based on the medical records, the mortality was attributed to the acute PE.

**Table 1:** Basic characteristics of patients of the three cohorts. sPESI = simplified pulmonary embolism severity index, eGFR = estimated glomerular filtration rate, BNP = brain natriuretic peptide.

	Trainings cohort	Validation cohort	External validation cohort
Total number of patients	339	169	186
Women (%)	58.5	55.3%	48.8
Age	64.9 ± 15.5	61.9 ± 10.2	64 ± 17
sPESI	1.3 ± 1.1	1.1 ± 1.0	2 ± 1
Wells Score	-	-	6 ± 2
eGFR (ml/min/1,73m^2^)	73.5 ± 39.1	70.5 ± 33.4	70 ± 28
NT-proBNP (pg/mL)	-	-	2112 ± 2594
D-dimer	6.5 ± 6.2	6.7 ± 5.5	13 ± 15

**Fig. 3:**
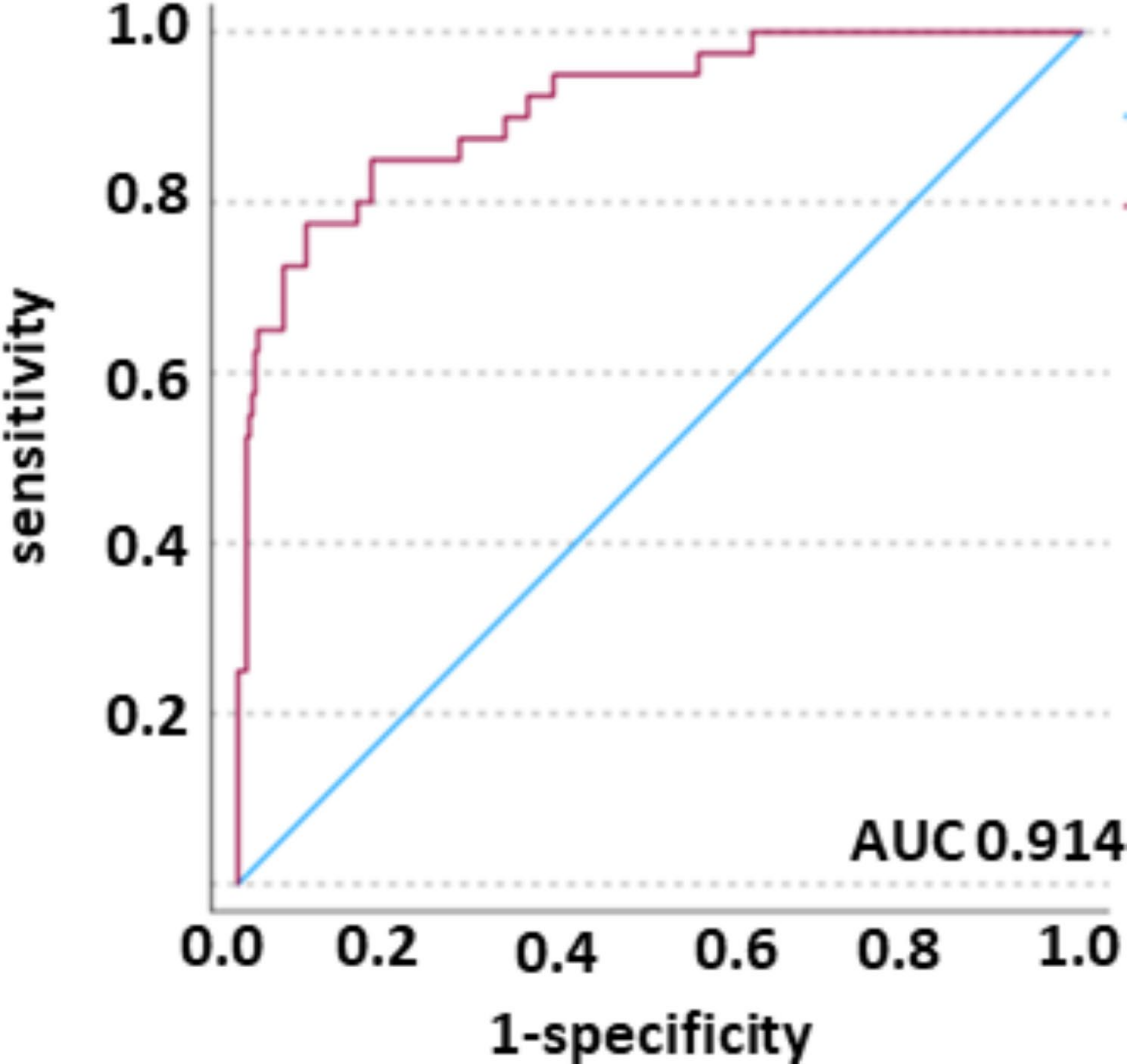
Predicted probability (red line), reference line (blue line). AUC was 0.914 (CI 0.866–0.963).

We started with analysis of features in a training cohort (*n* = 339) in order to decipher the best fitting model. We characterized the trainings cohort.The feature model incorporates a feature combination of 13 features of seven different image filters. Table 2 provides descriptions of the investigated texture features. Here, we fitted the features into the regression model, as the selected features represented the most important feature filter class, both quantitatively and by rank. Therefore, for the models, we decided to use the best fitting feature combination by applying logistic regression. The analysis revealed, that the model was best in discriminating the dichotomous variables. While the overall correctness of the models predicting mortality was comparable, the model performed better in discriminating the true positive cases (patients, who were correctly assigned to the “died” category). The model was superior in predicting mortality than rolling the dice (prediction of true positive 72.5%). 54.5% of the variance in the outcome, so whether patients died or survived, can be explained by the predictors used in the model (Nagelke R Square). Hosmer- Lemeshow test was non- significant in all models tested. The overall correctness of prediction of mortality is 88.1 (30-day mortality) and 79.7% (7-day mortality), respectively. The resulting Receiver Operator Characteristic (ROC) curves for the feature signature model is presented in Fig. 3. The ROC yields an area-under-the-curve (AUC) value of 0.914 with a 95% confidence interval (CI) of 0.866–0.963.

**Table 2:** Radiomic feature classes description. Selected features for the prediction of mortality are listed.

Features	Class of feature	description
log-sigma-2-0-mm-3D_firstorder_Skewness	First Order Features	First-order statistics describe the distribution of voxel intensities within the image region defined by the mask through commonly used and basic metrics. Skewness measures the asymmetry of the distribution of values about the Mean value. Depending on where the tail is elongated and the mass of the distribution is concentrated, this value can be positive or negative.
log-sigma-4-0-mm-3D_firstorder_Uniformity		Uniformity is a measure of the sum of the squares of each intensity value. This is a measure of the homogeneity of the image array, where a greater uniformity implies a greater homogeneity or a smaller range of discrete intensity values.
wavelet-LHL_firstorder_InterquartileRange		Here P25 and P75 are the 25th and 75th percentile of the image array, respectively.
squareroot_glszm_HighGrayLevelZoneEmphasis	Gray Level Size Zone Matrix (GLSZM) Second order features	A Gray Level Size Zone (GLSZM) quantifies gray level zones in an image. A gray level zone is defined as the number of connected voxels that share the same gray level intensity. Contrary to GLCM and GLRLM, the GLSZM is rotation independent, with only one matrix calculated for all directions in the ROI. HighGrayLevelZoneEmphasis measures the distribution of the higher gray-level values, with a higher value indicating a greater proportion of higher gray-level values and size zones in the image.
log-sigma-4-0-mm-3D_glrlm_GrayLevelNonUniformityNormalized	Gray Level Size Zone Matrix (GLSZM) Second Order Features	GrayLevelNonUniformityNormalized measures the variability of gray-level intensity values in the image, with a lower value indicating a greater similarity in intensity values. This is the normalized version of the GLN formula.
log-sigma-2-0-mm-3D_glszm_GrayLevelNonUniformityNormalized		
log-sigma-2-0-mm-3D_gldm_GrayLevelVariance		GrayLevelVariance measures the variance in gray level intensities for the zones.
wavelet-HHH_gldm_LowGrayLevelEmphasis		LowGrayLevelEmphasis measures the distribution of lower gray-level size zones, with a higher value indicating a greater proportion of lower gray-level values and size zones in the image.
wavelet-HHL_glszm_LargeAreaHighGrayLevelEmphasis		LargeAreaHighGrayLevelEmphasis measures the proportion in the image of the joint distribution of larger size zones with higher gray-level values.
wavelet-LHH_glrlm_RunVariance	Gray Level Run Length Matrix (GLRLM) Second Order Features	A Gray Level Run Length Matrix (GLRLM) quantifies gray level runs, which are defined as the length in number of pixels, of consecutive pixels that have the same gray level value. RunVariance is a measure of the variance in runs for the run lengths.
wavelet-LLH_glcm_SumSquares	Gray Level Co-occurrence Matrix (GLCM) Second Order Features	A Gray Level Co-occurrence Matrix (GLCM) of a size describes the second-order joint probability function of an image region constrained by the mask. Sum of Squares or Variance is a measure in the distribution of neigboring intensity level pairs about the mean intensity level in the GLCM.
wavelet-HLH_glcm_ClusterShade	Gray Level Co-occurrence Matrix (GLCM) Second Order Features	Cluster Shade is a measure of the skewness and uniformity of the GLCM. A higher cluster shade implies greater asymmetry about the mean.
wavelet-LLH_gldm_SmallDependenceEmphasis	Gray Level Dependence Matrix (GLDM) Second Order Features	A Gray Level Dependence Matrix quantifies gray level dependencies in an image. A gray level dependency is defined as a the number of connected voxels within distance δ that are dependent on the center voxel. A measure of the distribution of small dependencies, with a greater value indicative of smaller dependence and less homogeneous textures.

**Fig. 4:**
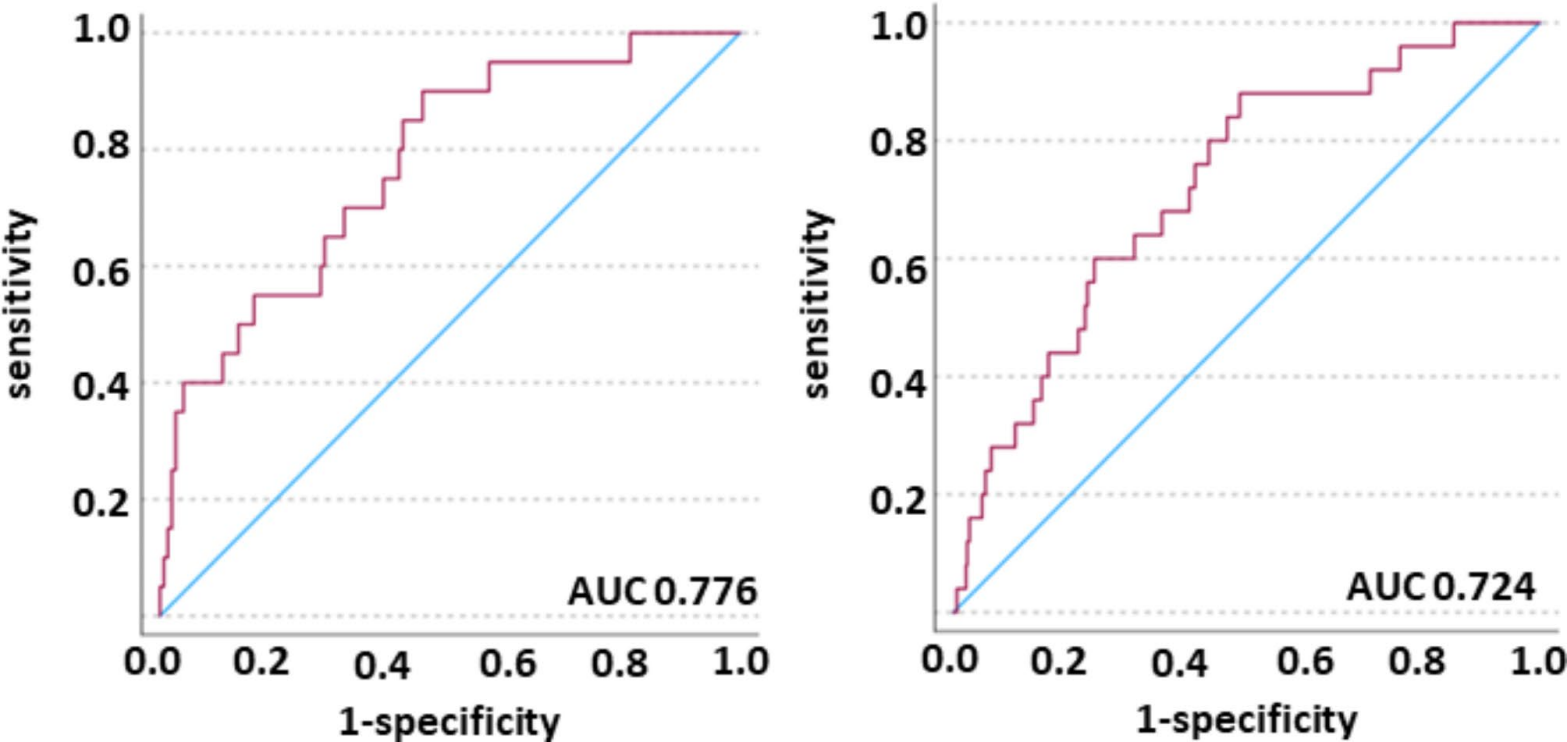
Validation cohort. Predicted probability (red line), reference line (blue line). Left: model 30-day mortality; right: model 7-day mortality. The AUC was 0.776 (CI 0.671–0.881) and 0.724 (CI 0.628–0.820), respectively.

**Fig. 5:**
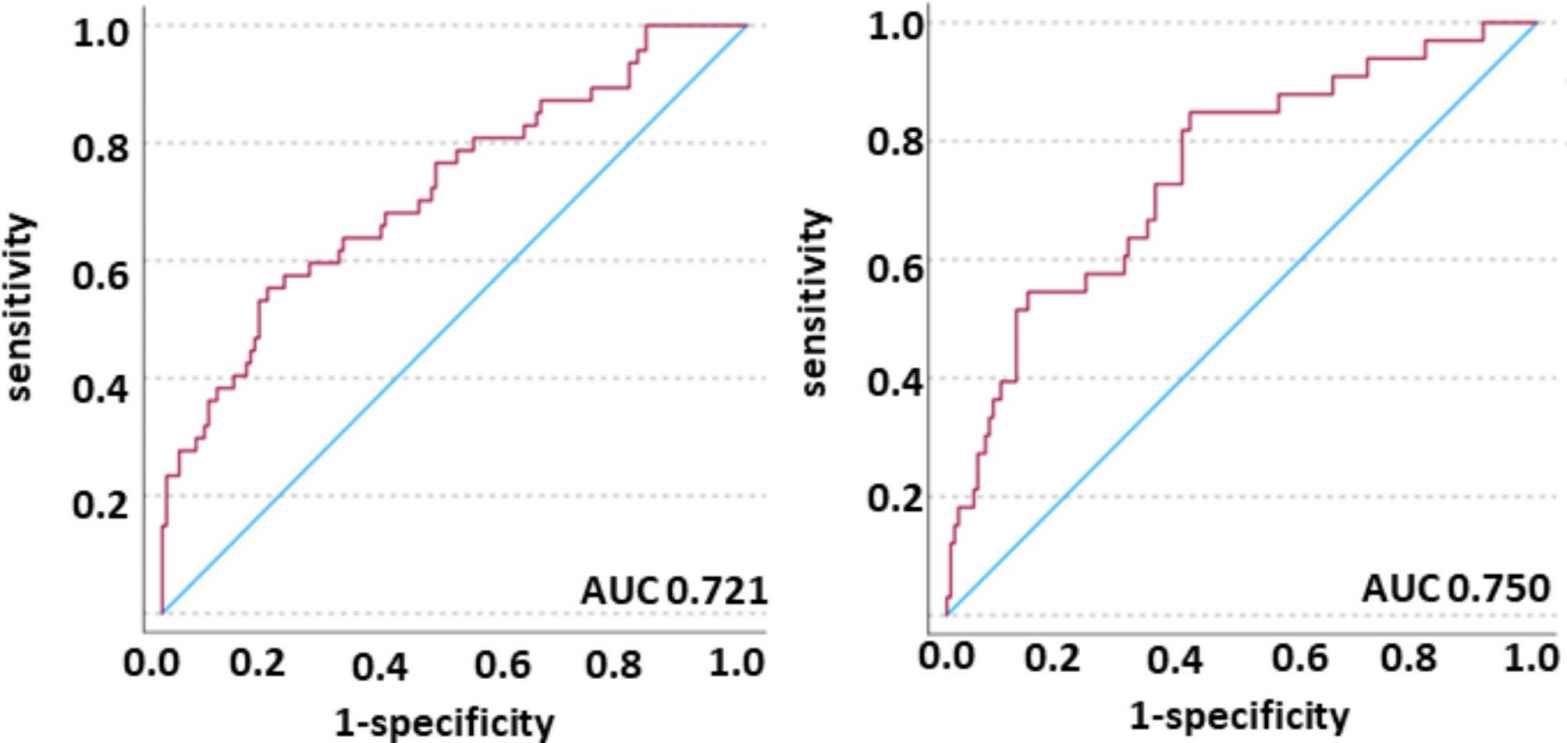
External validation cohort. Predicted probability (red line), reference line (blue line). Left: model 30-day mortality; right: model 7-day mortality. The AUC was 0.721 (CI 633-0.808) and 0.750 (CI 0.657–0.842), respectively.

**Fig. 6:**
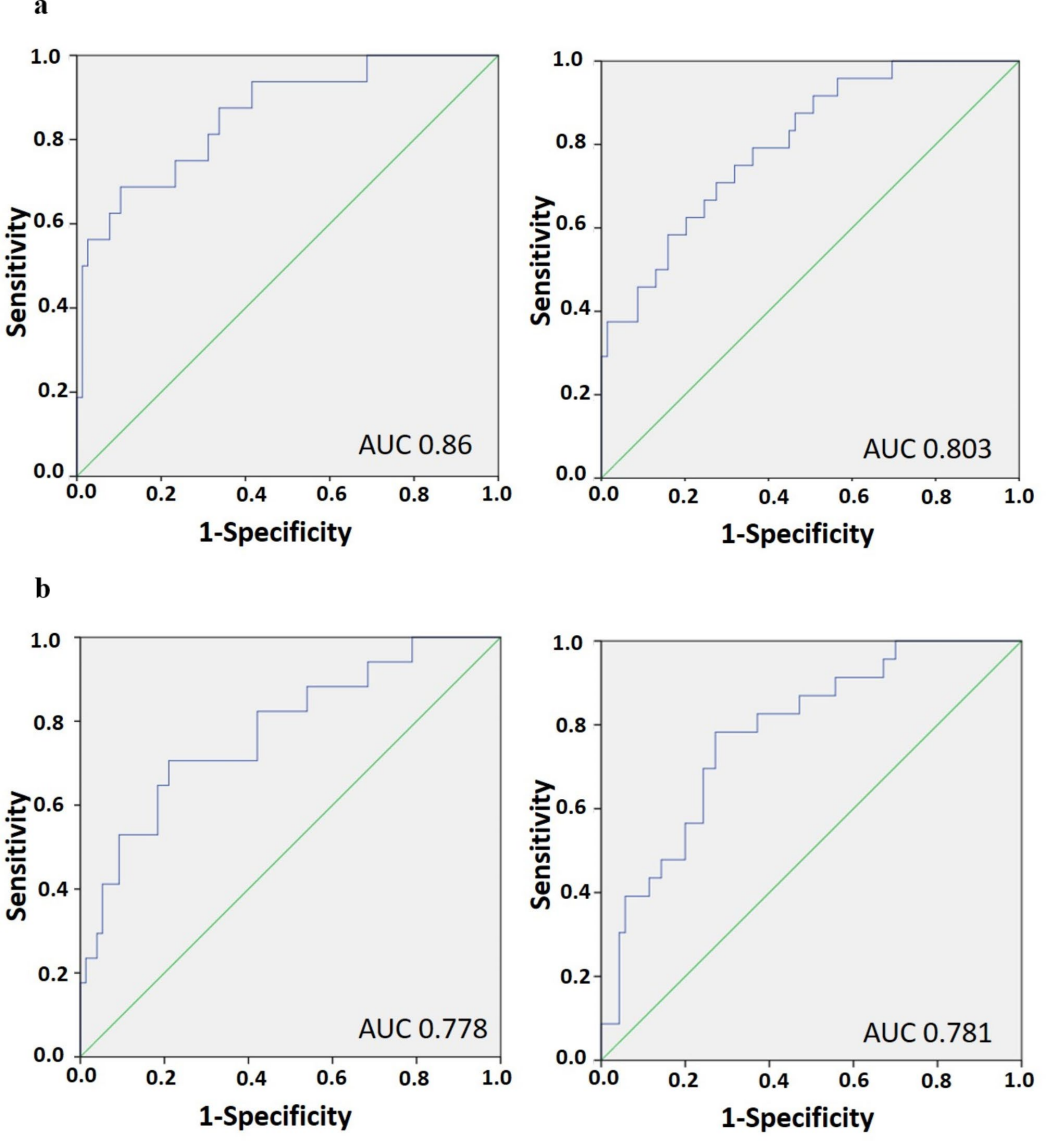
**a, b**. External validation cohort. Predicted probability (blue line), reference line (green line). **a**, left: model 7-day mortality in men (AUC 0.860 (CI 0.756–0.962)); right: model 30-day mortality in men (AUC 0.803 (CI 0.705-0.900)). **b**, left: model 7-day mortality in women (AUC 0.778 (CI 0.651–0.905)); right: model 30-day mortality in women (AUC 0.781 (CI 0.679–0.884)).

Finally, we validated the built model in a validation cohort (*n* = 169) in order to test accuracy of our model. We observed an AUC of 0.776 (CI 0.671–0.881, 30-day mortality and of 0.724 (CI 0.628–0.820, 7-day mortality) (Fig. 4). To corroborate these results in an independent external validation, we fitted the characterized model to a validation cohort (*n* = 186) in order to test the reliability of our model in an independent cohort. We observed an AUC of 0.721 (CI 0.633–0.808) in 30-day mortality and an AUC of 0.750 (CI 0.657–0.842) in 7-day mortality (Fig. 5). Aiming to analyze gender-specific effects of radiomic features, we split the independent cohort in men and women and observed a superiority of mortality prediction in men over women (AUC 0.860 (CI 0.756–0.962) in 7-day mortality and AUC 0.803 (CI 0.705-0.900) in 30-day mortality versus AUC 0.778 (CI 0.651–0.905) in 7-day mortality and 0.781 (CI 0.679–0.884) in 30-day mortality, respectively; Fig. 6a, b).

**Fig. 7:**
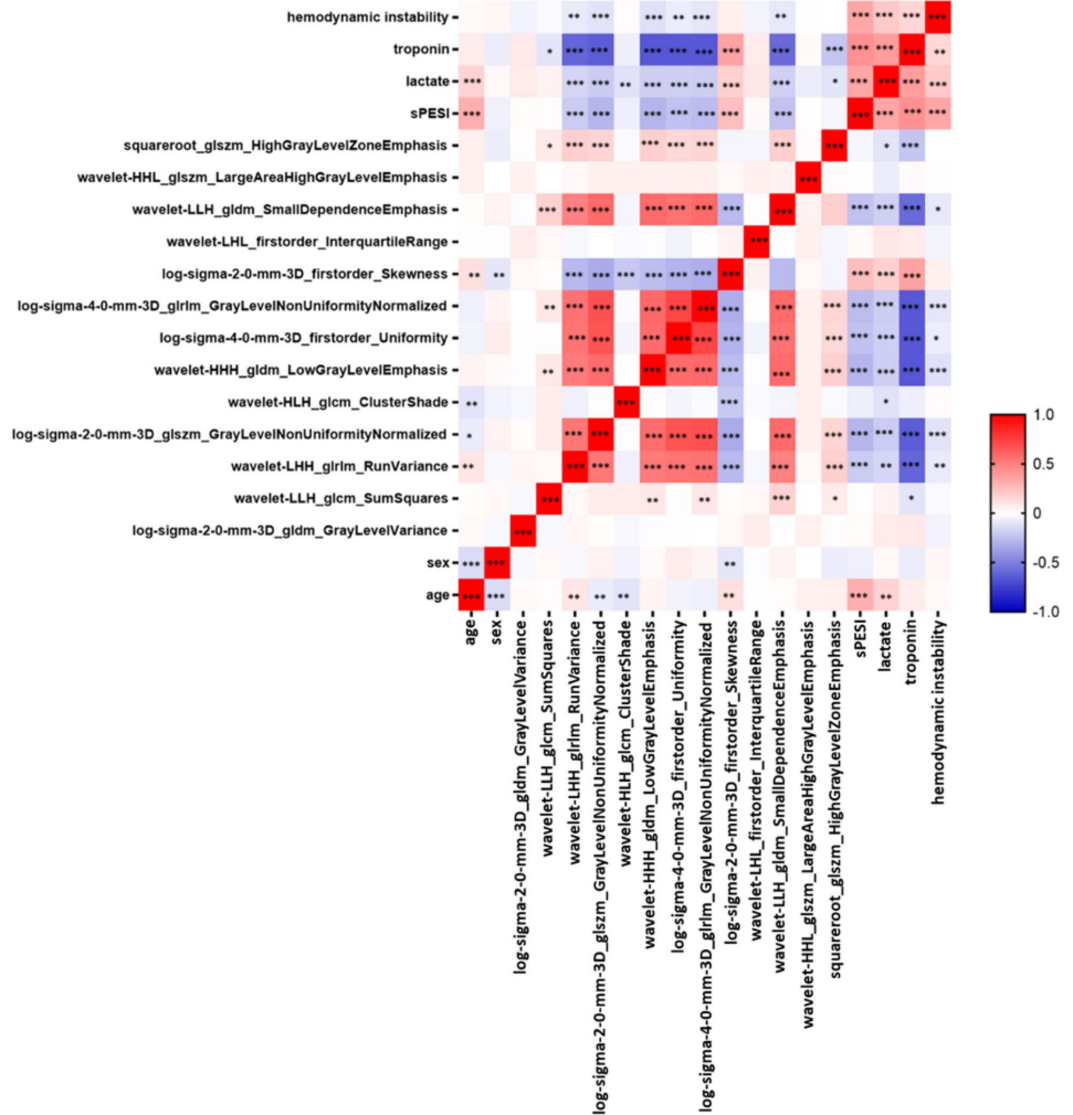
Correlation between age, sex, sPESI, lactate levels, troponin levels, hemodynamic instability and radiomics features. The heatmap represents Spearman correlations between relative changes in variables. Colors indicate the correlation coefficient, ranging from blue (− 1,0) to white (0,0) to red (1,0). Stars represent significant *p*-values: * *p* < 0.05; ** *p* < 0.01; *** *p* < 0.001.

We next correlated the signature features with levels of troponin, lactate, hemodynamic instability, age and sPESI (Fig. 7). Hemodynamic instability and lactate levels showed no relevant correlations with the radiomics features. Troponin levels correlated strongly with the features wavelet-LHH_glrlm_RunVariance (*r* = -0.61, *p* < 0.001), log-sigma-2-0-mm-3D_glszm_GrayLevelNonUniformityNormalized (*r* = -0.64, *p* < 0.001), wavelet-HHH_gldm_LowGrayLevelEmphasis (*r* = -0.66, *p* < 0.001), log-sigma-4-0-mm-3D_firstorder_Uniformity (*r* = -0.66, *p* < 0.001), log-sigma-4-0-mm-3D_glrlm_GrayLevelNonUniformityNormalized (*r* = -0.66, *p* < 0.001) and with wavelet-LLH_gldm_SmallDependenceEmphasis (*r* = -0.59, *p* < 0.001). Finally, the sPESI score correlated with age (*r* = 0.31, *p* < 0.001), log-sigma-2-0-mm-3D_glszm_GrayLevelNonUniformityNormalized (*r* = -0.29, *p* < 0.001) and wavelet-HHH_gldm_LowGrayLevelEmphasis (*r* = -0.29, *p* < 0.001).

## Discussion

Radiomics analysis is an emerging field of diagnosis especially in the context of oncology. Recently, radiomics got is in special focus of research in cardiology [14], aiming to improve prognosis of the diseases. We hypothesized that radiomics analysis of CT images of EAT may have a prognostic value in patients with APE. This is the first study addressing the prognostic role of radiomics based parameters of EAT in APE. EAT is a type of visceral fat surrounding the myocardium and visceral pericardium.

Complex interactions between EAT and the myocardium occur due to the fact, that EAT represents a metabolically active organ and influences anatomical and electrical remodeling of the heart. For instance, EAT induces fatty atrial infiltration [15]. Furthermore, EAT secretes pro-inflammatory and pro-fibrotic mediators [15, 16]. So far, according to Zhang et al., EAT shows an increased expression of leptin in patients with coronary artery disease [17]. Furthermore, leptin expression in EAT is an independent risk factor for coronary atherosclerosis [17]. EAT volume correlates significantly with serum concentration of irisin, adiponectin and leptin, which affects the myocardium [18]. EAT thickness correlates with inflammatory mediators like C-reactive protein and interleukin 6 [19]. 

Therefore, EAT plays an important role in cardiac homeostasis and may have a predictive role in APE. We hypothesize that EAT provoke a “chronic metabolic heart damage”. Metabolically damaged heart is not able to tolerate the APE.

As mentioned, cardiac dysfunction plays a key role in APE [20, 21]. The current risk assessment comprises troponin level as an independent predictor of short- and long-term outcomes in patients with PE [20]. Moreover, common prognostic factors are signs of right ventricular dysfunction (RVD) on CT and/or echocardiography [22]. CT-detected dilation of the right ventricle and reflux into the inferior vena cava are strong predictors of 30-day mortality in patients with APE [6, 21, 22]. 

We hypothesized that radiomics features of EAT predict 30-day mortality in APE. In fact, some studies indicate that EAT attenuation/density is more important than volume [8, 23]. For example, Pandey et al. showed that epicardial fat attenuation, but not volume, predicts obstructive coronary artery disease and high-risk plaque features in patients with atypical chest pain [8]. Presumably, changed EAT texture parameters may be associated with mortality in APE. Corroborating the idea, that the deeper structure analysis of EAT in APE using radiomics feature models is of prognostical value, we show, that radiomics features predict mortality in PAE with a high accuracy. Our data reveals an overall percentage of correct prediction of 90.5% in model 1 and 88.1% in model 2 in the validation cohort. This finding suggests that radiomics parameters may reflect deep changes or remodeling and thus indirectly also metabolic activity of EAT. Hypothetically, remodeled EAT may synthesize extensively or store more biochemical substances and can hence alter myocardial function. This phenomenon was already described in patients with cardiac arrhythmia [11, 24]. So far, in patients with aortic stenosis, radiomics features of EAT can identify patients at risk of developing postoperative atrial fibrillation [24]. Furthermore, a radiomics model shows good performance in predicting myocardial ischemia [25], whereas EAT radiomics signatures can predict recurrence of atrial fibrillation [26]. 

Independently of possible pathomechanisms, we postulate that CT based radiomics values of EAT play a highly predictive role and, therefore, they can be suggested as novel biomarkers in patients with APE.

Additionally, we could demonstrate a superior mortality prediction using radiomic features in men versus women. Future studies with larger cohorts are needed in order to verify our findings. Additionally, we did observe a better prediction in men as compared to both sexes merged, indicating that due to a small cohort size after splitting of sex, we might obtain a slight overfit in our model.

Our study has several limitations. Besides the retrospective nature, the manual segmentation of EAT is potentially error-prone and could be improved by additional readers or further automation processes. In future, we plan to extend our approach to a larger multi-center study. Lastly, the prediction models might further be limited by the unequal cohort split into the outcome categories „died“ versus „survived“, as the former category only included 1/10 of the study cohort. However, the present study is the first investigation about the prognostic role of radiomics parameters of EAT in patients with APE. Moreover, in this work, also an external validation of the radiomics-based models was performed.

In conclusion, we identified a radiomics signature of EAT that are strongly associated with 7-day and 30-day mortality in patients with APE. This could be shown to be linked by the associations between CT radiomics features and laboratory findings, here troponin levels and sPESI suggesting cardiac injury.

## Data Availability

No datasets were generated or analysed during the current study.

## Abbreviations

APE: Acute pulmonary embolism
AUC: Area under the curve
CTPA: Computer tomographic pulmonary angiography
EAT: Epicardial adipose tissue
sPESI: Simplified Pulmonary Embolism Severity Index
